# Mobile Health for Tuberculosis Management in South India: Is Video-Based Directly Observed Treatment an Acceptable Alternative?

**DOI:** 10.2196/11687

**Published:** 2019-04-03

**Authors:** Anil A Kumar, Ayesha De Costa, Arundathi Das, GA Srinivasa, George D'Souza, Rashmi Rodrigues

**Affiliations:** 1 St John's Research Institute St John's National Academy of Health Sciences Bangalore India; 2 Public Health Sciences Karolinska Institutet Stockholm Sweden; 3 Rajiv Gandhi University of Health Sciences Bangalore India; 4 Department of Chest Medicine St John's Medical College St John's National Academy of Health Sciences Bangalore India; 5 Department of Community Health St John's Medical College St John's National Academy of Health Sciences Bangalore India; 6 The Wellcome Trust/DBT India Alliance Hyderabad India

**Keywords:** medical informatics, tuberculosis, mHealth, adherence, mobile phone, reminder, SMS, voice call, DOT, vDOT, video DOT

## Abstract

**Background:**

With the availability of low-cost mobile devices and the ease of internet access, mobile health (mHealth) is digitally revolutionizing the health sector even in resource-constrained settings. It is however necessary to assess end-user perceptions before deploying potential interventions.

**Objective:**

This study aimed to assess the mobile phone usage patterns and the acceptability of mobile phone support during care and treatment in patients with tuberculosis (TB) in South India.

**Methods:**

This exploratory study was conducted at an urban private tertiary care teaching hospital and nearby public primary-level health care facilities in Bangalore, South India. We recruited 185 patients with TB through consecutive sampling. Subsequent to written informed consent, participants responded to an interviewer-administered pretested questionnaire. The questionnaire included questions on demographics, phone usage patterns, and the benefits of using of mobile phone technology to improve health outcomes and treatment adherence. Frequency, mean, median, and SD or interquartile range were used to describe the data. Bivariate associations were assessed between demographics, clinical details, phone usage, and mHealth communication preferences using the chi-square test and odds ratios. Associations with a *P* value ≤.20 were included in a logistic regression model. A *P* value of <.05 was considered significant.

**Results:**

Of the 185 participants, 151 (81.6%) used a mobile phone, and half of them owned a smartphone. The primary use of the mobile phone was to communicate over voice calls (147/151, 97.4%). The short message service (SMS) text messaging feature was used by only 66/151 (43.7%) mobile phone users. A total of 87 of the 151 mobile phone users (57.6%) knew how to use the camera. Only 41/151 (27.2%) mobile phone users had used their mobile phones to communicate with their health care providers. Although receiving medication reminders via mobile phones was acceptable to all participants, 2 participants considered repeated reminders as an intrusion of their privacy. A majority of the participants (137/185, 74.1%) preferred health communications via voice calls. Of the total participants, 123/185 (66.5%) requested reminders to be sent only at specific times during the day, 22/185 (11.9%) suggested reminders should synchronize with their prescribed medication schedule, whereas 40/185 (21.6%) did not have any time preferences. English literacy was associated with a preference for SMS in comparison with voice calls. Most participants (142/185, 76.8%) preferred video-based directly observed treatment when compared with in-person directly observed treatment.

**Conclusions:**

Although mobile phones for supporting health and treatment adherence were acceptable to patients with TB, mHealth interventions should consider language, mode of communication, and preferred timing for communication to improve uptake.

## Introduction

World tuberculosis (TB) surveillance estimates that 10 million people are either diagnosed or relapse with TB every year [1]. With a case fatality rate of 16%, TB is one of the most frequent causes for death from a single infectious agent, second only to HIV/AIDS [1]. Furthermore, the emergence of HIV infection in 1983, led to a resurgence in TB, making TB-HIV coinfection a threat of greater significance. India contributes 27% to the world’s burden of TB, the highest among the 10 high TB burden countries globally [1,2].

To address the burden of TB globally, the World Health Organization introduced the directly observed treatment, short-course (DOTS) strategy in 1992 [3]. DOTS comprises the following 5 elements: (1) political will for TB control, (2) case finding through quality diagnostics, (3) regular supply of antitubercular treatment (ATT), (4) short-course chemotherapy, which is the directly observed treatment (DOT) and, (5) a reliable TB information system [3]. DOTS was initiated in India in 1993, in a phased manner, through the Revised National Tuberculosis Control Program (RNTCP) [4]. The RNTCP provides ATT at no cost to patients with TB. However, as it implements DOTS, patients are required to visit a health care provider and swallow their medication under observation [4]. The alternative is for patients with TB to avail treatment through the private health care sector, at a cost. It is estimated that twice the number of patients with TB are treated in the private health care sector when compared with the public sector [5].

Given that TB requires 6 months of treatment with up to 4 antitubercular drugs, ensuring treatment success is a challenge, both from the patients’ and health care providers’ perspective. The need to stay motivated throughout the treatment along with a high pill burden, medication side effects, poverty, stigma, and discrimination, serve as barriers to treatment adherence [6-8]. In addition, forgetfulness and HIV coinfection can influence adherence negatively [7,9]. Along with these patient-associated barriers, health care system–related factors, such as ATT stockouts and unfavorable attitudes of health care providers toward patients with TB, also play a role [8,10-13].

Most Indian literature on ATT nonadherence reflects the proportions of patients lost to follow-up once initiated on ATT. The global TB report 2018 indicates that only 69% of Indian patients initiated on ATT are treated successfully; the rest either fail the treatment or are lost to follow-up or succumb to the illness [14]. Literature indicates that loss to follow-up with ATT in India ranges from 6% to 44% [15-18], whereas proportions of patients interrupting treatment for more than 1 month range from 14% to 50% [7,19].

Nonadherence to ATT has led to the emergence of drug-resistant strains of TB, which are resistant to either a single drug (monoresistance) or several drugs (multidrug resistance, MDR) or are extremely drug-resistant TB [20]. MDR TB treatment is associated with a higher financial burden, longer duration of treatment, and lower treatment success rates [21]. Given this, ensuring early diagnosis and treatment of TB minimizes the pill burden, making treatment regimens shorter, cheaper, and easier to comply with.

India has approximately 1.18 billion wireless subscribers, including mobile phone users [22]. Such a high wireless user base makes the use of mobile phones for health care delivery inevitable. Mobile phone reminders, such as voice calls and short message service (SMS) text messaging, to improve adherence to ATT have shown mixed results [23,24]. However, mobile reminders are known to improve clinic attendance [25]. Furthermore, mobile health (mHealth) [26] interventions have led to better retention of patients with TB when compared with historical cohorts [27]. A study from Lesotho, Africa, indicates that 92% of HIV/TB patients found SMS reminders for medications acceptable [28]. However, a randomized controlled trial from Pakistan found that SMS did not significantly improve treatment outcomes compared with a control group [29]. *Photovoice*, an app that used video recordings from patients cured of TB to promote ATT adherence and outcomes in Pune, India, showed better outcomes in patients exposed to the intervention [30]. In addition, mobile video-based directly observed treatment (vDOT), an alternative to conventional in-person DOT [31], holds promise, given the high mobile phone penetration and wireless users, especially in the Indian context [32].

Many mobile phone apps that are in use for TB are health care provider–centric and aid in either data collection or referral of patients with TB [33,34]. Given that few mobile apps for management of TB exist, mHealth for TB and its treatment is underexplored [34]. Furthermore, there is a paucity of information in the Indian context regarding the use of mobile phone interventions for TB. We therefore decided to explore the acceptability of mobile phone apps and the type of apps patients with TB would prefer. Such information is expected to support the development of patient-centric mobile phone apps for TB in the Indian context.

## Methods

### Study Site

The participants for this cross-sectional, exploratory study were recruited through consecutive sampling from both private and public sector health care facilities in Bangalore, Karnataka, India. The private facility was St John’s Medical College Hospital (SJMCH), Bangalore. This is a 1250 bedded, nonprofit, tertiary care teaching hospital that caters to patients largely from the South Indian states of Karnataka, Andhra Pradesh, and Tamil Nadu. The public health facilities involved were urban health centers in the vicinity of SJMCH in Bangalore. These public health facilities implement national health programs and provide health care at no cost to all those in need.

### Diagnosis and Treatment of Tuberculosis at St John’s Medical College Hospital (Private Health Care Facility)

Patients suspected of clinically active TB are subjected to microbiological and/or radiological tests for confirmation. Newly diagnosed patients with TB are started on a Category I ATT comprising isoniazid (H), rifampicin (R), pyrazinamide, and ethambutol for 6 months. This includes an initial 2 months of an intensive phase of treatment with all the 4 drugs, followed by 4 months of a continuation phase of treatment with 2 drugs (H and R). Patients with relapse or drug resistance receive more complex ATT regimens involving injectable medications. ATT at the hospital is available either through (1) the DOT center, a public-private initiative that enables no-cost treatment through the RNTCP or (2) for a cost through the hospital’s pharmacy. Patients follow up with physicians every month routinely for health appraisals that include clinical examination, monitoring adverse effects, or prescription refills.

### Diagnosis and Treatment of Tuberculosis at Public Facilities

Diagnostic protocols at the public health care facilities are similar to those at SJMCH. However, all patients diagnosed with TB and treated at public health care facilities receive treatment only through the RNTCP. All patients within the RNTCP are expected to receive DOT.

### Participants and Data Collection

Between February 2016 and December 2017, 185 patients with TB aged between 18 and 60 years, receiving treatment at the study sites, were enrolled in the study. Of the participants enrolled, 159/185 (85.9%) received ATT at SJMCH, whereas 26/185 (14.1%) received ATT at public health care facilities. Both newly diagnosed patients with TB and those already receiving ATT were included in the study. Patients who were seriously ill or those who did not understand the purpose of the study were excluded.

Subsequent to written informed consent, trained research assistants administered a questionnaire in the local language to the study participants. The questionnaire obtained basic sociodemographic information from the participants along with information regarding (1) the basic functionality of their mobile phones, (2) acceptability of delivering adherence support via mobile phones, and (3) the type of mobile phone intervention acceptable to them, such as SMS, voice call, interactive voice response system, or vDOT.

### Data Analysis

Data were analyzed using IBM-SPSS version 24. Frequencies, means, medians, SDs, and interquartile ranges were used to describe the data. The outcome variables studied were (1) the preference for voice calls compared with SMS reminders and (2) the preference for in-person DOT compared with vDOT. Some categorical variables that had multiple categories were converted into binary variables. Bivariate associations were assessed between demographics, clinical details, phone usage, and the preference-based outcome variables using the chi-square test and odds ratio (OR). Unadjusted logistic regression was used to derive OR for variables with more than 2 categories. Bivariate associations with a *P* value ≤.20 were included in an adjusted logistic regression model. Associations with *P* values <.05 were considered significant.

### Ethics Statement

Ethical clearance for the study was obtained from the Institutional Ethics Committee, St John’s Medical College, Bangalore, India. Written informed consent was obtained from all participants after providing them with study-related information, either verbally or in writing, before administering the questionnaire.

## Results

### Overview

A total of 185 patients with TB participated in the study. The mean age of the participants was 35.25 (SD 11.59) years. Of the participants, 114/185 (61.6%) were males, and 121/185 (65.4%) resided in an urban area. There were 44/185 (23.8%) participants on in-person DOT, 45/185 (24.3%) on self-administered treatment, and 96/185 (51.9%) participants for whom treatment was yet to be initiated. The demographic characteristics of the patients are presented in Table 1.

Clinically, 98/185 (53.0%) patients had pulmonary TB, and 159/185 (85.9%) were newly diagnosed patients with TB on category I ATT.

### Ownership of Mobile Phones

Of the 185 participants, 151/185 (81.6%) used a mobile phone. Among these 144/151 (95.4%) owned the phone, and 85/151 (56.3%) had used mobile phones for 6 years or more. Of those who owned mobile phones, 65/144 (45.1%) owned smartphones, and the rest (79/144, 54.9%) owned basic phones. The major reasons cited for not owning a phone included not needing a phone (19/34, 56%), inability to use a mobile phone (8/34, 24%), and financial constraints (3/34, 9%). Only 7/144 (4.9%) mobile phone users reported using a phone shared with other family members. Men were 4 times as likely as women to own mobile phones (unadjusted OR 3.816; 95% CI 1.747-8.338). Significant factors associated with phone ownership included education and a monthly income of 500 INR or more (Table 2).

**Table 1:** Demographic profile of study participants (N=185).

Variables		Total (n=185)	Female (n=71)	Male (n=114)	*P* value
**Age (years)**					
	Median (IQR^a^)	32 (26-45)	30 (24-45.5)	33 (27-44)	—^b^
	≥32, n (%)	97 (52.4)	37 (52)	58 (50.9)	Referent^c^
	<32, n (%)	88 (47.6)	34 (48)	56 (49.1)	.20
**Marital status, n (%)**					
	Married	123 (66.5)	45 (63)	78 (68.4)	Referent
	Single	62 (33.5)	26 (37)	36 (31.6)	.48
**Residence, n (%)**					
	Rural	64 (34.6)	20 (28)	44 (38.6)	Referent
	Urban	121 (65.4)	51 (72)	70 (61.4)	.15
**Education status, n (%)**					
	No formal education	40 (21.6)	17 (24)	23 (20.2)	Referent
	Formal education^d^	145 (78.4)	54 (76)	91 (79.8)	.55
**Literate in English, n (%)**					
	No	116 (62.7)	43 (61)	73 (64.0)	Referent
	Yes	69 (37.3)	28 (39)	41 (36.0)	.64
**Employment status, n (%)**					
	Not gainfully employed	76 (41.1)	49 (69)	27 (23.7)	Referent
	Gainfully employed	109 (58.9)	22 (31)	87 (76.3%)	<.001
**Monthly income (INR^e^)**					
	Median (IQR)	5000 (0-12000)	0 (0-5000)	9000 (2000-15000)	—
	≥5000, n (%)	97 (52.4)	71 (100)	59 (51.8)	Referent
	<5000, n (%)	88 (47.6)	0 (0)	55 (48.2)	<.001
**Type of patient, n (%)**					
	New patient^f^	159 (85.9)	64 (90)	95 (83.3)	Referent
	Others^g^	26 (14.1)	7 (10)	19 (16.7)	.20
**Type of TB^h^**, **n (%)**					
	Pulmonary	98 (52.9)	32 (45)	66 (57.9)	Referent
	Extrapulmonary	87 (47.1)	39 (55)	48 (42.1)	.09
**Microscopy (TB bacilli), n (%)**					
	Negative	100 (54.1)	47 (66)	53 (46.5)	Referent
	Positive	85 (45.9)	24 (34)	61 (53.5)	.009
**Treatment phase, n (%)**					
	Intensive	159 (85.9)	65 (92)	94 (82.5)	Referent
	Continuation	26 (14.1)	6 (8)	20 (17.5)	.08
**Treatment category, n (%)**					
	Category I	164 (88.6)	65 (92)	99 (86.8)	Referent
	Others	21 (11.4)	6 (8)	15 (13.2)	.33
**Treatment observation, n (%)**					
	In-person directly observed treatment.	44 (23.8)	16 (22)	28 (24.6)	Referent
	Self-administered treatment	45 (24.3)	14 (20)	31 (27.2)	.60
	Not initiated	96 (51.9)	41 (58)	55 (48.2)	.48
**Recruitment, n (%)**					
	Public health care facilities	26 (14.1)	10 (14)	16 (14.0)	Referent
	St John’s Medical College Hospital (private)	159 (85.9)	61 (86)	98 (86.0)	.99

^a^IQR: interquartile range.

^b^Not applicable.

^c^Referent: reference category.

^d^Formal education: this category includes middle school and above.

^e^1 INR (Indian Rupee)=0.014 US $, November 2018.

^f^New patient: a patient newly diagnosed with tuberculosis.

^g^Others: treatment after loss to follow-up or retreatment.

^h^TB: tuberculosis.

**Table 2:** Access to mobile phones and its association with demographic characteristics (N=185).

Variables		Mobile phone not used (n=34), n (%)	Mobile phone used (n=151), n (%)	Unadjusted OR^a^ (95% CI)	Adjusted^b,c^ OR (95% CI)
**Sex, n (%)**					
	Female	22 (31)	49 (69)	Referent^d^	—^e^
	Male	12 (10.5)	102 (89.5)	3.816 (1.747-8.338)	—
**Education status, n (%)**					
	No formal education	14 (35)	26 (65)	Referent	Referent
	Formal education	20 (13.8)	125 (86.2)	3.365 (1.508-7.513)	2.623 (1.118-6.153)
**Literate in English, n (%)**					
	No	28 (24.1)	88 (75.9)	Referent	Referent
	Yes	6 (9)	63 (91)	3.341 (1.306-8.546)	—
**Employment status, n (%)**					
	Not gainfully employed	25 (33)	51 (67)	Referent	—
	Gainfully employed	9 (8.3)	100 (91.7)	5.447 (2.367-12.531)	—
**Monthly income (Indian Rupee), n (%)**					
	<5000	28 (32)	60 (68)	Referent	Referent
	≥5000	6 (6)	91 (94)	7.078 (2.765-18.120)	6.288 (2.428-16.290)
**Treatment observation, n (%)**					
	In-person directly observed treatment	8 (18)	36 (82)	Referent	—
	Self-administered treatment	11 (24)	34 (76)	0.687 (0.247-1.913)	—
	Not initiated	15 (16)	81 (84)	1.200 (0.467-3.083)	—
**Recruitment center, n (%)**					
	Public health care facility	6 (23)	20 (77)	Referent	—
	St John’s Medical College Hospital (private)	28 (17.6)	131 (82.4)	1.404 (0.517-3.813)	—

^a^OR: odds ratio.

^b^Logistic regression model *P* value<.001 (Forward stepwise [conditional] method); Nagelkerke *R*^2^: 0.215 (step 2); −2 Log-likelihood: 150.230 (step 2).

^c^Only variables retained in the final regression model have an adjusted OR.

^d^Referent: reference category.

^e^Not applicable.

### Basic Functionality of Mobile Phones

Of the participants who used a mobile phone, 66/151 (43.7%) used the SMS feature, 72/151 (47.7%) used the alarm function, whereas 87/151 (57.6%) knew how to use the camera on their phone for photography and/or videography. Of those who used the alarm on the phone, only 2/73 (3%) used the alarm as a medication reminder.

Participants less than 32 years of age (adjusted OR 2.314, 95% CI 1.068-5.025) or those literate in English (adjusted OR 8.678, 95% CI 4.019-18.740) were more likely to use the SMS feature than their counterparts. In addition, those who were single (unadjusted OR 2.793, 95% CI 1.479-5.263), residing in an urban area (unadjusted OR 3.493, 95% CI 1.695-7.195), or formally educated (unadjusted OR 15.012, 95% CI 3.489-64.591) were more likely to use the SMS compared with those who were married, were from a rural area, or were not formally educated, respectively (Table 3).

### Preferred Mobile Phone Interventions for Management of Tuberculosis

**Figure 1:**
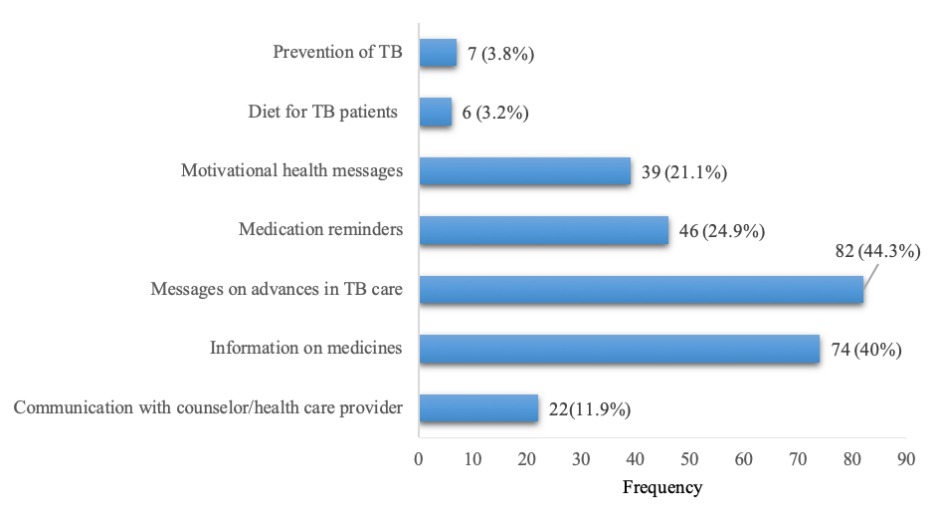
Type of health information requested over mobile phone (N=185). TB: tuberculosis.

Of the 185 participants, 182 (98.4%) agreed to receive health information on their mobile phones. Topics that the participants preferred included information on available medications, advances in TB management, and medication reminders. Participants also requested communication with health care provider, motivational health messages, specific diet, and prevention of TB as additional features (Figure 1).

In response to specific queries about the preferred mode of health communication, 137/185 (74.1%) of the participants chose voice calls over the SMS (40/185, 21.6%). In addition, 8/185 (4.3%) participants preferred either. Most of those (43/48, 90%) who preferred the SMS requested to receive them in English. On the contrary, most of those who preferred voice calls (127/137, 92.7%) requested for communication in regional languages. Of the 185 participants, 78 (42.2%) chose to receive reminders as often as they required to take their medication, whereas the rest preferred reminders either once a week or less frequently. Similarly, with regard to the timing of the reminders, 123/185 (66.5%) preferred reminders at specific times, whereas 22/185 (11.9%) preferred reminders just before they took their medication. The remaining 40/185 (21.6%) participants were willing to receive reminders *anytime* as they were *free at home*. Overall, 183/185 (98.9%) of the participants did not perceive the reminders as an intrusion of their privacy.

Preference for voice calls was significantly associated with age, marital status, literacy in English, type of TB, and the ability to use camera on their phones (Table 4).

Most of the study participants preferred vDOT over the conventional in-person DOT. This preference was associated with being male, residing in an urban area, and being formally educated. Other factors such as literacy in English, the ability to use SMS and phone camera, although associated with preference for vDOT in bivariate analyses, were not found associated with this preference in an adjusted logistic regression model. Clinical characteristics were not associated with the preference for the mode of communication or adherence monitoring strategy (vDOT or in-person DOT; Table 5).

**Table 3:** Use of text messaging and its association with demographic characteristics (N=185).

Variables		SMS^a^ not used^b^ (n=119), n (%)	SMS used (n=66), n (%)	Unadjusted OR^c^ (95% CI)	Adjusted^d,e^ OR (95% CI)
**Sex, n (%)**					
	Female	45 (63)	26 (37)	Referent^f^	—^g^
	Male	74 (64.9)	40 (35.1)	0.936 (0.505-1.734)	—
**Age (years)**					
	≥32, n (%)	78 (80)	19 (20)	Referent	Referent
	<32, n (%)	41 (47)	47 (53)	4.716 (2.450-9.009)	2.314 (1.068-5.025)
**Marital status, n (%)**					
	Married	89 (72.4)	34 (27.6)	Referent	—
	Single	30 (48)	32 (52)	2.793 (1.479-5.263)	—
**Residence, n (%)**					
	Rural	52 (81)	12 (19)	Referent	—
	Urban	67 (55.4)	54 (44.6)	3.493 (1.695-7.195)	—
**Education status, n (%)**					
	No formal education	38 (95)	2 (5)	Referent	—
	Formal education	81 (55.9)	64 (44.1)	15.012 (3.489-64.591)	—
**Literate in English, n (%)**					
	No	99 (85.3)	17 (14.7)	Referent	Referent
	Yes	20 (29)	49 (71)	14.268 (6.865-29.654)	8.678 (4.019-18.740)
**Monthly income (Indian Rupee), n (%)**					
	<5000	64 (73)	24 (27)	Referent	—
	≥5000	55 (57)	42 (43)	2.036 (1.098-3.776)	—
**Treatment observation, n (%)**					
	In-person directly observed treatment	28 (64)	16 (36)	Referent	—
	Self-administered treatment	33 (73)	12 (27)	0.636 (0.258-1.569)	—
	Not initiated	58 (60)	38 (40)	1.147 (0.548-2.398)	—
**Recruitment center, n (%)**					
	Public health care facility	18 (69)	8 (31)	Referent	—
	St John’s Medical College Hospital (private)	101 (63.5)	58 (36.5)	1.292 (0.529-3.157)	—

^a^SMS: short message service.

^b^Comprised those who did not use SMSs as they did not have a phone and those who had a phone but did not use the feature.

^c^OR: odds ratio.

^d^Logistic regression model *P* value<.001 (Forward stepwise [Conditional] method); Nagelkerke *R*^2^: 0.445 (step 3); −2 Log-likelihood: 168.595 (step 3).

^e^Only variables retained in the final regression model have an adjusted OR.

^f^Referent: reference category.

^g^Not applicable.

**Table 4:** Preference for an intervention and its association with clinical and demographic characteristics (N=185).

Variables		Prefer SMS^a^ (n=48), n (%)	Prefer voice call (n=137), n (%)	Unadjusted OR^b^ (95% CI)	Adjusted^c,d^ OR (95% CI)
**Sex, n (%)**					
	Female	20 (28)	51 (72)	Referent^e^	—^f^
	Male	28 (24.6)	86 (75.4)	1.204 (0.616-2.354)	—
**Age (years)**					
	<32, n (%)	40 (46)	48 (55)	Referent	Referent
	≥32, n (%)	8 (8)	89 (92)	9.271 (4.017-21.396)	4.129 (1.557-10.947)
**Marital status, n (%)**					
	Single	30 (48)	32 (52)	Referent	Referent
	Married	18 (14.6)	105 (85.4)	5.469 (2.700-11.076)	2.934 (1.172-7.346)
**Residence, n (%)**					
	Rural	8 (13)	56 (88)	Referent	—
	Urban	40 (33.1)	81 (66.9)	0.289 (0.126-0.665)	—
**Education status, n (%)**					
	No formal education	1 (3)	39 (97)	Referent	—
	Formal education	47 (32.4)	98 (67.6)	0.053 (0.007-0.401)	—
**Literate in English, n (%)**					
	No	12 (10.3)	104 (89.7)	Referent	Referent
	Yes	36 (52)	33 (48)	0.106 (0.049-0.227)	0.265 (0.108-0.652)
**Employment status, n (%)**					
	Not gainfully employed	17 (22)	59 (78)	Referent	—
	Gainfully employed	31 (28.4)	78 (71.6)	0.725 (0.367-1.433)	—
**Monthly income (Indian Rupee), n (%)**					
	<5000	16 (18)	72 (82)	Referent	—
	≥5000	32 (33)	65 (67)	0.451 (0.227-0.898)	—
**Type of patient, n (%)**					
	New patient	44 (27.7)	115 (72.3)	Referent	—
	Others	4 (15)	22 (85)	2.104 (0.686-6.453)	—
**Type of TB^g^, n (%)**					
	Extrapulmonary	28 (32)	59 (68)	Referent	Referent
	Pulmonary	20 (20)	78 (80)	1.851 (0.950-3.597)	3.205 (1.290-7.936)
**Microscopy (TB bacilli), n (%)**					
	Negative	33 (33)	67 (67)	Referent	—
	Positive	15 (18)	70 (82)	2.299 (1.146-4.611)	—
**Treatment phase, n (%)**					
	Intensive	40 (25.2)	119 (74.8)	Referent	—
	Continuation	8 (31)	18 (69)	0.756 (0.305-1.873)	—
**Treatment category, n (%)**					
	Category I	45 (27.4)	119 (72.6)	Referent	—
	Others	3 (14)	18 (86)	2.269 (0.638-8.075)	—
**Treatment status, n (%)**					
	In-person directly observed treatment	15 (34)	29 (66)	Referent	—
	Self-administered treatment	6 (13)	39 (87)	3.362 (1.163-9.721)	—
	Not initiated	27 (28)	69 (72)	1.322 (0.615-2.843)	—
**Recruitment center, n (%)**					
	Public health care facility	8 (31)	18 (69)	Referent	—
	St John’s Medical College Hospital (private)	40 (25.2)	119 (74.8)	1.322 (0.534-3.274)	—
**Access to phone, n (%)**					
	No	4 (12)	30 (88)	Referent	—
	Yes	44 (29.1)	107 (70.9)	0.324 (0.108-0.975)	—
**SMS use, n (%)**					
	No	13 (10.9)	106 (89.1)	Referent	—
	Yes	35 (53)	31 (47)	0.109 (0.051-0.230)	—
**Camera use, n (%)**					
	No	9 (9)	89 (91)	Referent	Referent
	Yes	39 (45)	48 (55)	0.124 (0.056-0.278)	0.243 (0.092-0.640)

^a^SMS: short message service.

^b^OR: odds ratio.

^c^Logistic regression model *P* value<.001 (Forward stepwise [conditional] method); Nagelkerke *R*^2^: 0.491; −2 Log-likelihood: 136.497.

^d^Only variables retained in the final regression model have an adjusted OR.

^e^Referent: reference category.

^f^Not applicable.

^g^TB: tuberculosis.

**Table 5:** Preference for video-based directly observed treatment to in-person directly observed treatment and its association with demographics, clinical details, and mobile phone usage characteristics.

Variables		Prefer DOT^a^ (n=43), n (%)	Prefer vDOT^b^ (n=142), n (%)	Unadjusted OR^c^ (95% CI)	Adjusted OR^d,e^ (95% CI)
**Sex, n (%)**					
	Female	26 (37)	45 (63)	Referent^f^	Referent
	Male	17 (14.9)	97 (85.1)	3.297 (1.627-6.680)	4.004 (1.846-8.683)
**Age (years)**					
	<32, n (%)	14 (16)	74 (84)	Referent	—^g^
	≥32, n (%)	29 (30)	68 (70)	0.444 (0.216-0.909)	—
**Marital status, n (%)**					
	Single	11 (18)	51 (82)	Referent	—
	Married	32 (26.1)	91 (73.9)	0.613 (0.285-1.319)	—
**Residence, n (%)**					
	Rural	22 (34)	42 (66)	Referent	Referent
	Urban	21 (17.4)	100 (82.6)	2.494 (1.241-5.014)	2.626 (1.197-5.765)
**Education status, n (%)**					
	No formal education	18 (45)	22 (55)	Referent	Referent
	Formal education	25 (17.2)	120 (82.8)	3.927 (1.841-8.376)	3.391 (1.492-7.709)
**Literate in English, n (%)**					
	Others	34 (29.3)	82 (70.7)	Referent	—
	English	9 (13)	60 (87)	2.764 (1.234-6.193)	—
**Employment status, n (%)**					
	Not gainfully employed	20 (26)	56 (74)	Referent	—
	Gainfully employed	23 (21.1)	86 (78.9)	1.335 (0.672-2.655)	—
**Monthly income (Indian Rupee), n (%)**					
	<5000	26 (30)	62 (70)	Referent	—
	≥5000	17 (18)	80 (82)	1.973 (0.984-3.956)	—
**Type of patient, n (%)**					
	New patient	37 (23.3)	122 (76.7)	Referent	—
	Others	6 (23)	20 (77)	1.011 (0.378-2.704)	—
**Type of TB^h^, n (%)**					
	Pulmonary	24 (24)	74 (76)	Referent	—
	Extrapulmonary	19 (22)	68 (78)	1.161 (0.585-2.305)	—
**Microscopy (TB bacilli) , n (%)**					
	Negative	25 (25)	75 (75)	Referent	—
	Positive	18 (21)	67 (78)	1.241 (0.623-2.473)	—
**Treatment phase, n (%)**					
	Intensive	40 (25.2)	119 (74.8)	Referent	—
	Continuation	3 (12)	23 (88)	2.577 (0.734-9.043)	—
**Treatment category, n (%)**					
	Category I	39 (23.8)	125 (76.2)	Referent	—
	Others	4 (19)	17 (81)	1.326 (0.421-4.175)	—
**Treatment observation, n (%)**					
	In-person DOT	13 (30)	31 (70)	Referent	—
	Self-administered	11 (24)	34 (76)	1.296 (0.507-3.315)	—
	Not initiated	19 (20)	77 (80)	1.699 (0.749-3.857)	—
**Recruitment center, n (%)**					
	Public health care facility	8 (31)	18 (69)	Referent	—
	St John’s Medical College Hospital (private)	35 (22.1)	124 (77.9)	1.575 (0.632-3.925)	—
**Access to phone, n (%)**					
	No	15 (44)	19 (56)	Referent	—
	Yes	28 (18.5)	123 (81.5)	3.468 (1.571-7.654)	—
**Short message service use, n (%)**					
	No	36 (30.3)	83 (69.7)	Referent	—
	Yes	7 (11)	59 (89)	3.656 (1.523-8.776)	—
**Camera use, n (%)**					
	No	29 (30)	69 (70)	Referent	—
	Yes	14 (16)	73 (84)	2.192 (1.069-4.492)	—

^a^DOT: directly observed treatment.

^b^vDOT: video-based directly observed treatment.

^c^OR: odds ratio.

^d^Final adjusted logistic regression model *P* value<.001 (Forward stepwise [conditional] method); Nagelkerke *R*^*2*^: 0.22; −2 Log-likelihood: 171.465.

^e^Only variables retained in the final regression model have an adjusted OR.

^f^Referent: reference category.

^g^Not applicable.

^h^TB: tuberculosis.

## Discussion

### Principal Findings

Barriers such as stigma, medication side effects, and transport to the health care facility can affect adherence to ATT. In this light, exploiting the pervasiveness of mobile phone technology to overcome these barriers and support medication adherence is a promising solution. Motivational health messages and customized medication reminders via mobile phones are some interventions designed to support adherence to ATT [24]. Although several studies have explored the use of mobile phone interventions for adherence support in chronic infectious diseases such as HIV and TB, not all have shown favorable results [29,35]. Factors such as the complexity, personalization, and mode of communication could affect acceptability, uptake, and success of mobile phone interventions. We therefore chose to explore the acceptability of mobile phone adherence support interventions for ATT and identify the characteristics of such interventions, which the patients with TB would prefer, before developing such an intervention.

In this study, most participants were willing to receive adherence reminders via mobile phones and did not consider such interventions as an intrusion of their privacy. Randomized controlled trials have shown the effectiveness of SMS reminders in HIV infection and malaria in sub Saharan Africa [36]. However, the effectiveness of mobile phone reminders for antiretroviral treatment support in India was questionable [35]. Nevertheless, given that treatment for TB is for 6 months compared with HIV infection where the treatment is lifelong, mHealth interventions for TB are likely to face lesser intervention fatigue and are worth exploring. Although patients with TB from Salem in Tamil Nadu state, South India, considered communication via mobile phones useful, they preferred in-person contact with health care providers. The study, however, did not assess the preferred mode or type of ATT adherence support [37].

A study from Lesotho, Africa, reported a high uptake of SMS interventions in HIV/TB patients [28]. On the contrary and consistent with other studies from South India, most of our study participants preferred voice calls in comparison with the SMS [38]. The young, the employed, and the educated participants were more likely to use the SMS for communication. These participants probably preferred reading an SMS text as opposed to answering a phone call as it saved time and attracted lesser attention when received. Given the limited literacy in English, the roman script was used for both English and regional languages in SMS communication; it is not surprising that SMS communication was less popular than voice calls both in this study and in the literature in the Indian context [37,38]. However, with the availability of regional language options in mobile phones, developing interventions in regional languages is an option worth exploring. Furthermore, given that some of the participants were not literate but could use the basic functionality on mobile phones, interventions that use videos and pictures with limited requirements for literacy are an option.

Asynchronous vDOT is an accepted alternative suggested to circumvent the barriers to in-person DOT [39]. Studies have shown that vDOT is more confidential, easy to use [40], and allows health care providers to efficiently monitor a larger number of patients at a distance when compared with in-person DOT [31]. Barriers to vDOT include interruption of data connectivity [31], loss or theft of phone [40], and an inability to confirm that medicine was actually taken in certain settings [41]. Over three-fourths of the participants preferred vDOT to in-person DOT, despite no experience with the intervention, citing vDOT as an optimal solution for saving time and money or minimizing hospital visits.

Few participants expressed concern with sending their video to the health care provider. Fear of disclosure of their illness to family, unknown people watching their videos, fear of the videos getting published via social media, and discomfort with video-recording themselves were reasons expressed for the concern. Therefore, counseling the beneficiaries of the measures taken to safeguard their videos along with reinforcing the importance of adherence to ATT is essential to ensure the uptake of vDOT. Smartphone apps, although nonintrusive, were found beneficial in the management of HIV infection despite their limited functionality [42].

In this light, the abundant features for patient-centric mHealth interventions for TB can be explored. Most of the participants suggested newer apps incorporating disease-related information and behavior change communication, which could be incorporated into existing or newer apps. The concept of *photovoice* [30], where patients cured of TB shared their treatment experiences and replaced health care personnel, can be considered an option. *Photovoice* can also be incorporated into vDOT for health education and communication with patients.

### Methodological Issues

Given that standard TB care in India is based on geographic location, most of the patients were from urban areas and therefore more representative of urban patients with TB. Also, as one-third of the patients received ATT through the RNTCP (at SJMCH or at public health care facilities), the study mirrors the public-private mix in TB care in India. Furthermore, as many of the study participants were newly diagnosed with limited treatment experience, their opinions are also likely to change with treatment. This, along with the limited experience of the participants with mobile phone interventions may mean that opinions may change with actual interventions. The limitations in estimating a sample size and the nonprobability sampling technique used may affect the generalizability of our findings. Nevertheless, the results of the study cannot be undermined as they inform patient-centric intervention design in contexts little exposed to mHealth interventions.

In addition, in terms of implementation reality, factors such as poverty, smartphone penetrance, internet access, and level of education necessary for using mobile phones will have to be addressed in an integrated manner to maximize the potential benefit of vDOT in ensuring treatment success in TB.

### Conclusions

This study sought to assess whether communication via mobile phones could be an acceptable form of health care delivery in the context of patients with TB. We found that adherence reminders and information disseminating apps were acceptable in the management of TB. Contrary to the popularity of SMS-based reminders elsewhere globally, most of the study participants preferred voice calls. Efficacy of mHealth interventions could be improved when components that enable the inclusion of all demographic groups are incorporated along with enabling customizations to individual needs. Given the popularity of voice calls, interventions should include a voice component along with various language options in the Indian context. Although facing interceptable barriers such as privacy and stigma, vDOT as an alternative to DOT appears to hold promise in the Indian context. The effectiveness of mobile phone apps such as vDOT may therefore be worth exploring in the Indian context, while ensuring privacy and confidentiality of the end user.

## Abbreviations

ATT: antitubercular treatment
DBT: Department of Biotechnology
DOT: directly observed treatment
DOTS: directly observed treatment, short-course
H: isoniazid
MDR: multidrug resistance
mHealth: mobile health
OR: odds ratio
R: rifampicin
RNTCP: Revised National Tuberculosis Control Program
SJMCH: St John’s Medical College Hospital
SMS: short message service
TB: tuberculosis
vDOT: video-based directly observed treatment
